# Postpartum-Onset Dermatomyositis Presenting With Malignant Pleural Effusion: A Case Report

**DOI:** 10.7759/cureus.101370

**Published:** 2026-01-12

**Authors:** Eesha Zainab, Hamdia Gul Aslam, Ahmad Atif, Muhammad Imran

**Affiliations:** 1 Department of Pulmonology, Allama Iqbal Medical College, Lahore, PAK; 2 Department of Pulmonology, Jinnah Hospital, Allama Iqbal Medical College, Lahore, PAK; 3 Department of Pathology, Allama Iqbal Medical College, Lahore, PAK

**Keywords:** case report, dermatomyositis, malignant pleural effusion, paraneoplastic syndrome, postpartum

## Abstract

Dermatomyositis (DM) is a rare autoimmune inflammatory myopathy with cutaneous and muscular manifestations. Malignancy is associated with up to one-third of cases in women; gynecological cancer is among the most frequent associations. Postpartum-onset DM is exceptionally rare, with only a few cases in the literature. We report a case of postpartum-onset DM complicated by malignant pleural effusion. A 40-year-old Pakistani woman, 15 days postpartum following a cesarean section, presented with progressive facial swelling, heliotrope rash, photosensitivity, and proximal muscle weakness. She also reported dysphagia, alopecia, and weight loss. A strong family history of cancer was noted. Electromyography and MRI confirmed an inflammatory myopathy. Subsequently, she developed dyspnea and cough. Imaging revealed a right-sided pleural effusion with pleural nodules. Pleural fluid cytology and biopsy confirmed metastatic adenocarcinoma. Immunohistochemistry was positive for PAX8 and cytokeratin-7, suggesting a gynecological origin, although no primary gynecological tumor was identified on imaging. Positron emission tomography (PET)-computed tomography (CT) and laparoscopic staging were not feasible due to financial and clinical constraints. The patient was started on prednisolone and azathioprine, which significantly improved her DM symptoms. For the malignancy, she began systemic chemotherapy. The pleural effusion was managed with chest tube drainage, pleurodesis, and thoracotomy. Despite treatment, her prognosis remained poor due to advanced metastatic disease. Postpartum-onset DM presenting with malignant pleural effusion is rare and may signal an occult malignancy. This case highlights the importance of early cancer screening in similar presentations, as well as the need for a coordinated, multidisciplinary approach to optimize patient management and outcomes.

## Introduction

Dermatomyositis (DM) is a rare autoimmune inflammatory myopathy characterized by distinctive cutaneous findings, including heliotrope rash and Gottron papules, and symmetrical proximal muscle weakness. In adults, it presents as a paraneoplastic syndrome in about 15-30% of cases and can often indicate an underlying, undiagnosed malignancy [1]. Among women, the most frequently associated primary tumors are gynecological malignancies, especially ovarian and breast cancers [2]. Although DM most commonly presents between ages 45 and 60, its occurrence during childbearing years is uncommon, accounting for approximately 14% of cases [3]. Postpartum-onset DM is exceptionally rare, with isolated reports proposing triggers such as hormonal changes or fetal antigen exposure during the peripartum period [4]. The present case describes a unique occurrence of postpartum dermatomyositis revealing metastatic adenocarcinoma that initially presented with malignant pleural effusion.

## Case presentation

**Figure 1:**
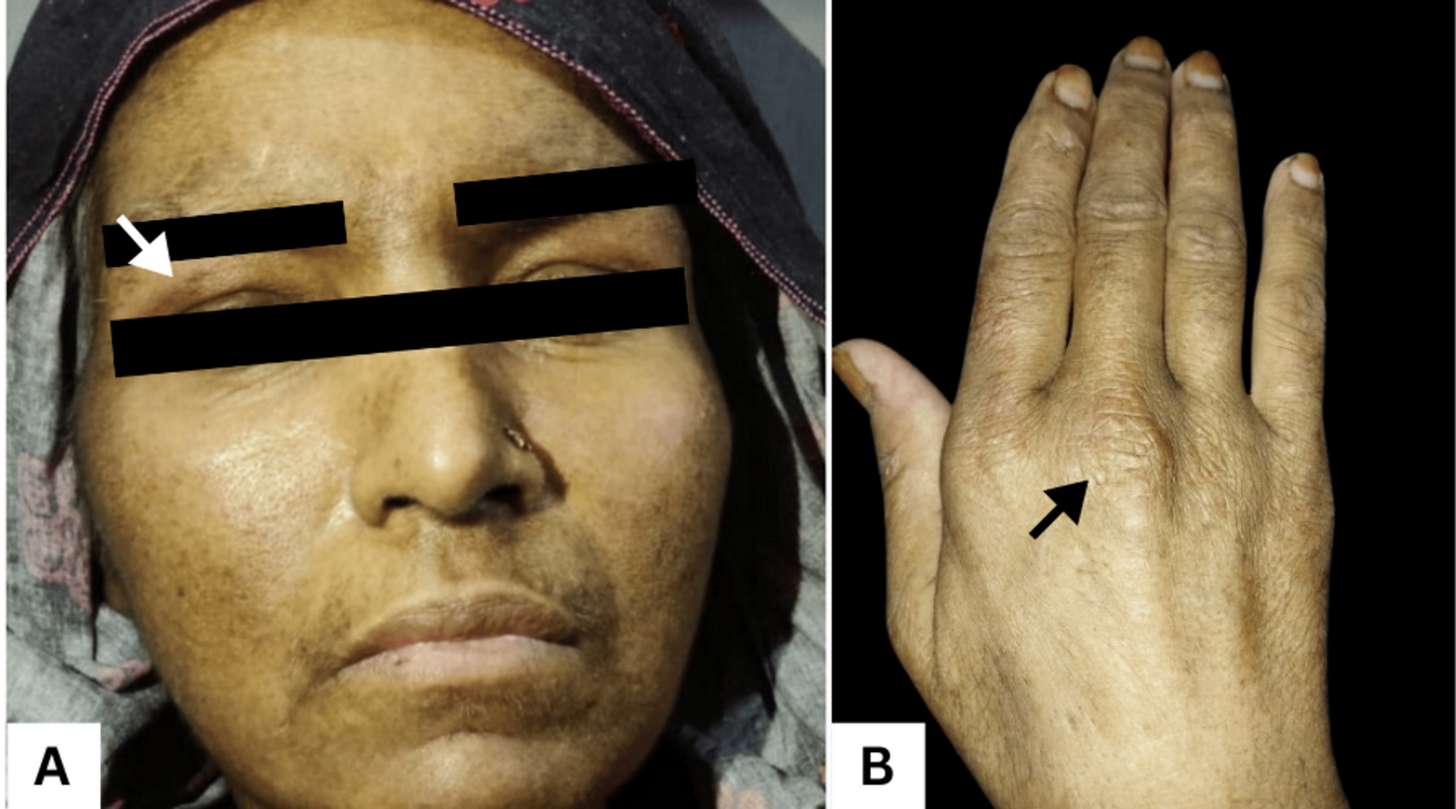
Classic cutaneous manifestations of dermatomyositis The facial photograph showing a heliotrope‑type violaceous erythema in the periorbital area (white arrow) with mild edema over the forehead and cheeks (A) and hand, revealing dry, wrinkled skin, with the black arrow showing a Gottron‑type papule (B), classic cutaneous findings of dermatomyositis.

A 40-year-old Pakistani woman, 15 days postpartum following an uncomplicated cesarean section, presented with sudden-onset facial and cervical swelling accompanied by an erythematous rash. The rash initially appeared on her knuckles, face, arms, chest, and thighs, later progressing to violaceous discoloration with photosensitivity (Figure 1). She also reported progressive proximal muscle weakness, dysphagia, diffuse alopecia, and arthralgias in the small joints of her hands and feet. A strong family history of malignancy was noted, with her mother, sister, and niece all having died from unspecified cancers.

Clinical and diagnostic findings

**Figure 2:**
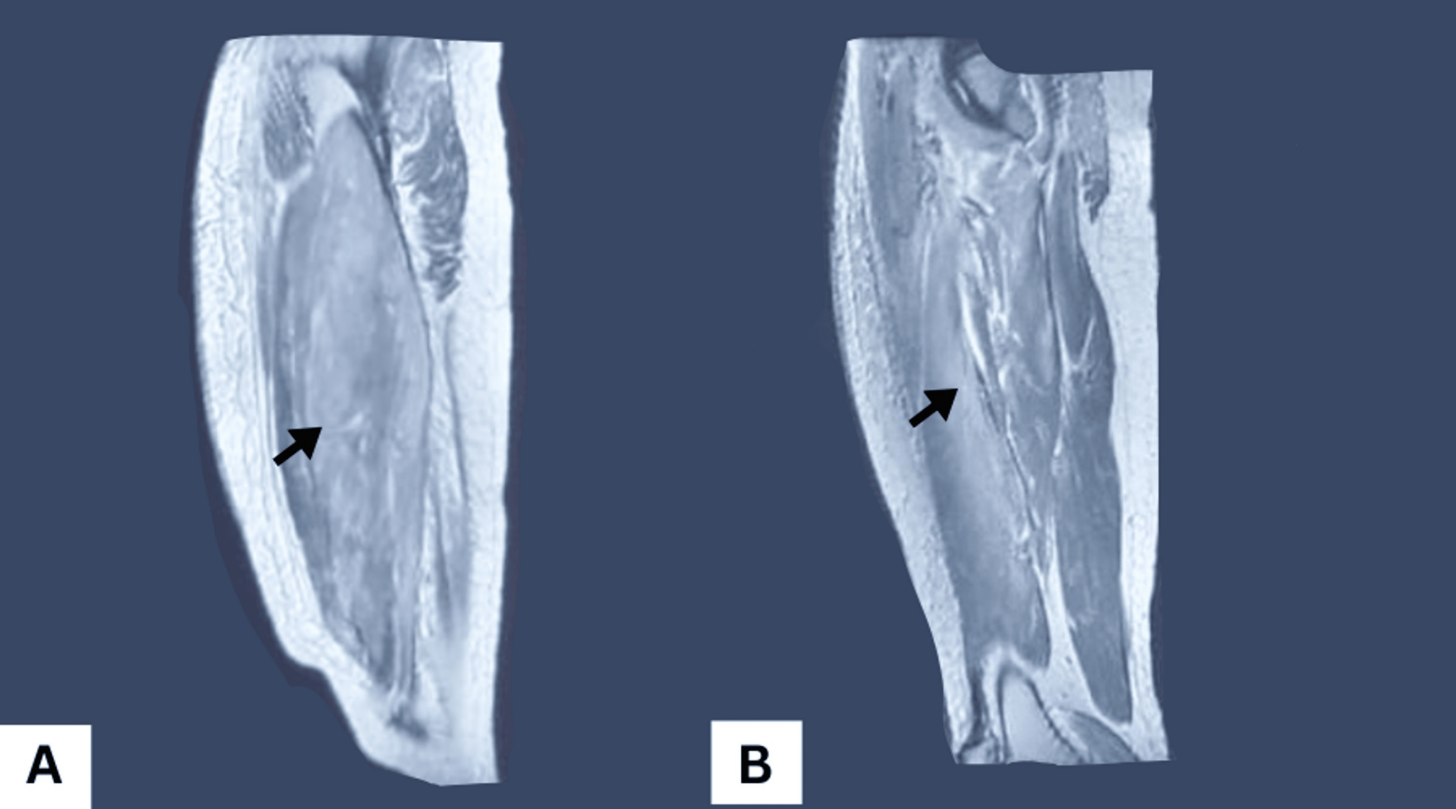
Sagittal T2-weighted fat-saturated thigh MRI Demonstrating diffuse muscle swelling in the anterior thigh, especially the quadriceps, extending into the posterior hamstring muscles with high signal intensity (black arrow) on PD‑FS images, indicating edema and inflammatory myopathy consistent with dermatomyositis.

Laboratory investigations revealed markedly elevated creatine kinase (CK), lactate dehydrogenase (LDH), and aldolase, consistent with inflammatory myopathy. Autoantibody panels, including ENA, were negative. Electromyography (EMG) demonstrated proximal myopathy, and MRI of the thighs showed swollen muscles with hyperintense signals on PD-FS sequences, supporting a diagnosis of inflammatory myositis (Figure 2). An incidental hepatic hydatid cyst, classified as Gharbi stage II, was also identified in segment VI of the right hepatic lobe on abdominal ultrasound.

Onset of respiratory symptoms and pleural effusion

**Figure 3:**
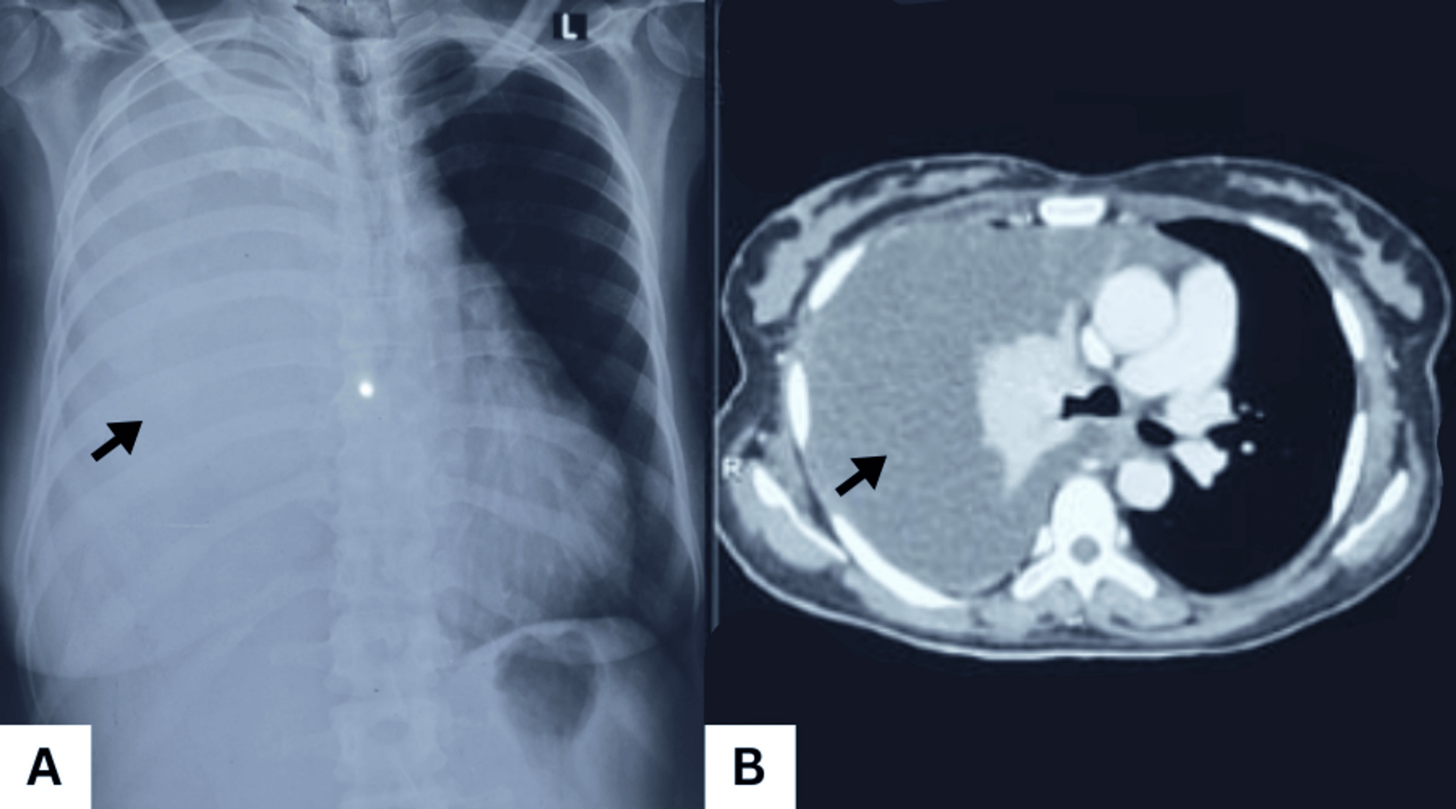
Chest X-ray (A) and CT chest (B) Showing white out right hemithorax with a shift of the mediastinum to the left side, suggestive of massive pleural effusion

Two weeks after DM onset, the patient developed progressive dyspnea, productive cough, and significant unintentional weight loss. Physical examination revealed absent breath sounds over the right hemithorax. Chest X‑ray and CT revealed a large right pleural effusion with pleural nodularity, raising suspicion for malignant involvement (Figure 3).

Pleural fluid and biopsy

**Figure 4:**
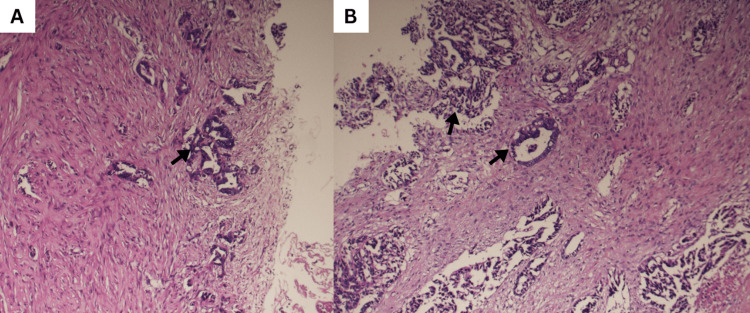
Pleural biopsy revealing a malignant adenocarcinoma A malignant epithelial proliferation forming irregular glandular and papillary structures (black arrows) infiltrating a markedly desmoplastic and fibrous stroma. Tumor cells exhibit high-grade features, including dense basophilic nuclei and scant cytoplasm, characteristic of invasive adenocarcinoma.

Cytology showed clusters of atypical cells with pleomorphic, hyperchromatic nuclei and a high N/C ratio. Pleural biopsy revealed metastatic adenocarcinoma, immunohistochemistry was PAX8 and CK7 positive, and negative for TTF‑1 and Napsin‑A, suggesting a non‑lung, likely gynecologic primary despite negative pelvic imaging (Figure 4). MRI of the brain and cervical spine was unremarkable aside from degenerative changes.

Management

Dermatomyositis was treated with prednisolone and azathioprine, resulting in symptomatic relief and a regain of her muscle strength. Pleural effusion required chest‑tube drainage and pleurodesis, with medical thoracoscopy and tube thoracostomy performed for diagnostic biopsy and management of the malignant effusion. Systemic chemotherapy, consisting of paclitaxel and carboplatin on a 21-day cycle, was initiated for the metastatic adenocarcinoma. The hepatic hydatid cyst was managed medically with albendazole. At her final follow-up prior to transfer, the patient’s dermatomyositis symptoms remained well-controlled on immunosuppressive therapy. However, due to significant financial constraints and the cumulative burden of diagnostic costs, she was discharged to continue her palliative chemotherapy in a local hospital. Her overall prognosis remained guarded due to the advanced metastatic adenocarcinoma.

## Discussion

DM is a rare idiopathic inflammatory myopathy with a prevalence of approximately 5 to 22 cases per 100,000 [4]. Only about 14% of cases occur during reproductive years, with the highest incidence typically observed between the ages of 50 and 60 years [3]. This case is exceptionally rare, as it represents the co-occurrence of two rare clinical phenomena: postpartum-onset DM and malignant pleural effusion. While DM has a well-recognized paraneoplastic association, its onset just two weeks following a cesarean section presents a critical diagnostic challenge, whether the condition was triggered by the postpartum immune rebound or if it was a paraneoplastic response to an occult malignancy.

Several aspects of this case strongly support paraneoplastic etiology. In adults, DM presents as a paraneoplastic syndrome in approximately 15% to 30% of cases [1]. In women, the condition is most frequently associated with ovarian and breast cancers. Clinical evidence indicates that ovarian cancer associated with DM often presents at an advanced stage (FIGO III or IV), with epithelial adenocarcinoma being the predominant histological type [5]. Furthermore, literature suggests that DM can precede a cancer diagnosis in approximately 40% of cases [6]. In this patient, the detection of malignant epithelial clusters in the pleural fluid combined with an immunohistochemistry (IHC) profile of PAX8 and CK7 positivity strongly tilts the diagnosis toward a paraneoplastic event, specifically suggesting a gynecological primary.

Conversely, the postpartum autoimmune hypothesis provides an alternative trigger. Pregnancy is characterized by relative immunosuppression, while the postpartum period involves a well-recognized “immune rebound” phase. This period of vulnerability is associated with the new onset or flare of various autoimmune diseases, potentially triggered by hormonal shifts and fetal antigen exposure [3]. While cases of postpartum-onset DM without concurrent malignancy exist, the presence of a malignant effusion in this patient makes a purely autoimmune etiology unlikely. It is more probable that the postpartum immune rebound facilitated the clinical expression of dermatomyositis that was fundamentally driven by the underlying metastatic adenocarcinoma.

This report is fundamentally limited by the inability to identify the primary tumor despite an extensive work-up. In lower-middle-income countries (LMIC) healthcare systems, financial constraints often limit access to advanced diagnostic modalities such as PET-CT or contrast-enhanced MRI, which might have localized the occult primary. Diagnostic efforts were restricted to conventional CT and ultrasound, which failed to reveal a definitive mass. Furthermore, histopathologic confirmation was limited by a basic IHC panel; the unavailability of crucial markers like CA-125 or gastrointestinal-specific markers (CK20/CDX2) restricted the ability to definitively rule out alternate primary sites. Procedural limitations, such as the inability to perform a diagnostic laparoscopy, further hindered definitive staging.

The challenges highlighted in this case underscore the need for optimized diagnostic pathways in resource-limited settings. To improve the diagnosis in settings with limited resources, a step-by-step testing approach is recommended. This begins with affordable, widely available tests like expanded IHC panels. Collaboration with larger reference laboratories can provide access to more specialized tools. Importantly, investing early in key diagnostics such as essential IHC and MRI is often more cost-effective in the long run than prolonged testing without clear results. This structured method is essential for accurate diagnosis and effective treatment in such complicated paraneoplastic disorders.

## Conclusions

This case illustrates the rare coexistence of postpartum‑onset dermatomyositis and a malignant pleural effusion, highlighting the diagnostic challenge of distinguishing a postpartum autoimmune disorder from a paraneoplastic phenomenon. It also underscores the importance of malignancy screening in adults with dermatomyositis, particularly in postpartum women, where immune and hormonal changes may unmask paraneoplastic disease. Malignant pleural effusion as the initial presentation of an occult malignancy in this context is rare and warrants a high index of suspicion. Early recognition, comprehensive cancer evaluation, and multidisciplinary management are essential to optimize outcomes in such complex presentations.
